# Flooding‐Driven Greenhouse Gas Emissions From Global Upland Croplands

**DOI:** 10.1002/advs.78387

**Published:** 2026-10-26

**Authors:** Shuilian Bi, Zhifeng Yan, Zhengkui Ge, Si‐Liang Li, Guirui Yu, Cong‐Qiang Liu, Hanqin Tian, Pete Smith

**Affiliations:** ^1^ Institute of Surface‐Earth System Science School of Earth System Science Tianjin University Tianjin China; ^2^ Critical Zone Observatory of Bohai Coastal Region Tianjin Key Laboratory of Earth Critical Zone Science and Sustainable Development in Bohai Rim Tianjin University Tianjin China; ^3^ Key Laboratory of Ecosystem Network Observation and Modeling Institute of Geographic Sciences and Natural Resources Research Chinese Academy of Sciences Beijing China; ^4^ Haihe Laboratory of Sustainable Chemical Transformations Tianjin China; ^5^ Department of Earth and Environmental Sciences Schiller Institute for Integrated Science and Society Boston College Chestnut Hill Massachusetts USA; ^6^ Institute of Biological and Environmental Sciences School of Biological Sciences University of Aberdeen Aberdeen UK

**Keywords:** emission factors, extreme rainfall, floods, greenhouse gases, nitrous oxide emissions, upland croplands

## Abstract

Greenhouse gas emissions from temporarily flooded upland croplands remain poorly characterized in the global greenhouse gas budget. We combined field measurements from 11 maize‐field sites temporarily flooded across the North China Plain with a global synthesis of 23 flooded upland cropland sites to quantify diffusive emissions. Flooded croplands release high fluxes of nitrous oxide, carbon dioxide, and methane, with emission factors exceeding relevant Intergovernmental Panel on Climate Change (IPCC) defaults for rice paddies and newly flooded lands. Extrapolating site‐level fluxes from limited observations over the estimated global area of flooded upland cropland and mean flooding duration yields a mean of 63.3 teragrams (Tg) of carbon dioxide‐equivalent (CO_2_‐eq) emissions per year during the standing‐water flooding periods, comprising 7.2 (1.7–17.2), 34.6 (9.0–83.7), and 21.5 (6.2–45.6) Tg from nitrous oxide, carbon dioxide, and methane, respectively. Temporarily flooded upland croplands are therefore identified as a previously overlooked component of the global greenhouse gas budget, providing an empirical basis for incorporating flood‐driven emissions into future agricultural inventories.

## Graphical abstract

Temporary flooding of upland croplands releases nitrous oxide, carbon dioxide, and methane. Field measurements combined with a global literature synthesis yield a global estimate of 63.3 teragrams of carbon dioxide equivalent per year for diffusive emissions from flooded upland croplands. These findings reveal an overlooked source in agricultural greenhouse gas budgets.

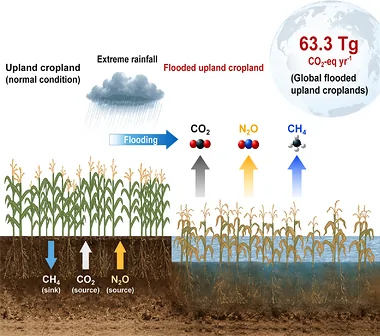

## 1 Introduction

Climate change is increasing the frequency and intensity of extreme rainfall events, exposing upland croplands worldwide to episodic flooding [1, 2, 3]. Unlike wetlands and rice paddies, upland cropland soils under typical non‐flooded conditions generally exhibit net nitrous oxide (N_2_O) emissions and weak atmospheric methane (CH_4_) uptake [4, 5, 6, 7]. Flooding rapidly disrupts these conditions by reducing oxygen availability, altering redox status, and mobilizing carbon and nitrogen substrates [8, 9, 10]. As a result, greenhouse gas (GHG) emissions from upland croplands may change substantially during flooding, but their magnitude and contribution to the global GHG budget remain poorly quantified.

Previous studies report contrasting GHG responses to flooding in upland croplands, reflecting the complex biogeochemical processes triggered by soil inundation. N_2_O and CO_2_ emissions can increase or decrease depending on oxygen availability, substrate supply and microbial activity [11, 12, 13, 14], whereas CH_4_ emissions generally increase because anaerobic conditions stimulate methanogenesis and suppress methane oxidation [15, 16]. The magnitude and direction of these responses are further influenced by substrate stoichiometry, soil and water pH, flooding depth and flooding duration [17, 18]. Existing evidence therefore remains fragmented across individual gases, environmental settings and regional case studies, preventing a comprehensive understanding of flooding‐driven GHG emissions at the global scale.

This evidence gap limits the representation of flooding‐driven GHG emissions in current agricultural inventories [2, 19]. Current Intergovernmental Panel on Climate Change (IPCC) guidelines provide emission factors for N_2_O and CH_4_ from flooded rice paddies [20, 21] and for CO_2_ from newly flooded lands such as reservoirs [19]. However, they do not explicitly account for temporarily flooded upland croplands. Flood‐prone uplands supporting maize, wheat, and soybean occupy extensive harvested areas worldwide, representing approximately 14% of the global harvested rice paddy area [22]. Their omission may lead to underestimation of agricultural GHG budgets and associated climate feedbacks. Addressing this gap is therefore essential for improving future GHG inventories and Earth system assessments.

Here, we combine field measurements from 11 flooded maize‐field sites across the North China Plain with a global synthesis of 23 flooded upland cropland sites (Figure 1). We quantify diffusive emissions of N_2_O, CO_2,_ and CH_4_, identify their environmental drivers and estimate global flood‐driven emissions from upland croplands (see Methods). We show that temporarily flooded upland croplands release high fluxes of all three gases and make a substantial contribution to global GHG emissions during flooding periods, identifying an overlooked source that is absent from current inventory frameworks.

**FIGURE 1:**
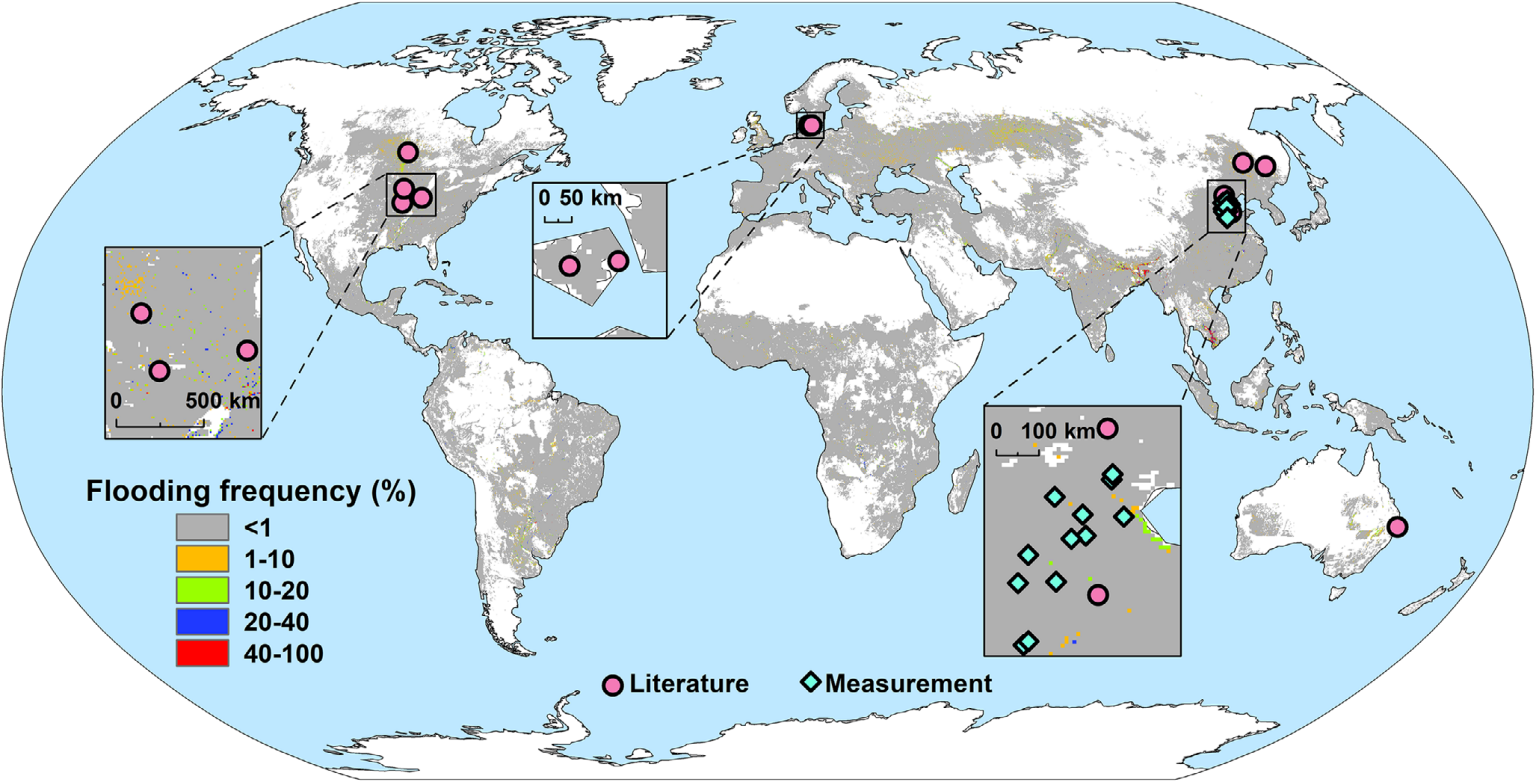
Global distribution of study sites and flood‐prone rainfed upland croplands. Blue diamonds indicate field sites sampled in this study and pink circles indicate sites compiled from the literature. Insets show site clusters in the United States Corn Belt, Northern Europe and the North China Plain. The background map shows the flooding frequency of rainfed upland croplands growing wheat, maize, barley, soybean and sugarcane during 2000–2018. Flooding frequency is expressed as the percentage of years in which cropland pixels were flooded. Pixels without target crops are shown as no data. Crop distribution was derived from Spatial Production Allocation Model (SPAM) 2020 v2.0, and flood extent from the Global Flood Database v1 [23].

## 2 Results

### 2.1 Flooded Croplands Emit High GHG Fluxes

Flooded upland croplands released high and variable diffusive GHG fluxes, indicating that episodic flooding can rapidly convert normally aerobic agricultural soils into substantial emission sources (Figure 2). Across the field measurements and global synthesis, N_2_O fluxes ranged from −18.9 to 2643 µmol m^−2^ d^−1^, with a mean of 284 ± 436 µmol m^−2^ d^−1^ (Figure 2A). The corresponding mean N_2_O emission factor was 0.027 ± 0.023 (Figure 2B). This value exceeds the IPCC default for flooded rice paddies and reported global means for maize and wheat [20, 24]. CO_2_ fluxes ranged from 5.0 to 4819 mmol m^−2^ d^−1^, with a mean of 1144 ± 1263 mmol m^−2^ d^−1^ (Figure 2C), well above the IPCC default for newly flooded lands such as reservoirs [19]. Diffusive CH_4_ fluxes ranged from 0.01 to 68.3 mmol m^−2^ d^−1^, with a mean of 11.1 ± 19.4 mmol m^−2^ d^−1^ (Figure 2D), comparable to or slightly exceeding values commonly reported for flooded rice paddies [21, 25]. These summary statistics were calculated from individual observations, with equal weight given to each observation (*n* = 79, 59, and 15 for N_2_O, CO_2_, and CH_4_, respectively).

**FIGURE 2:**
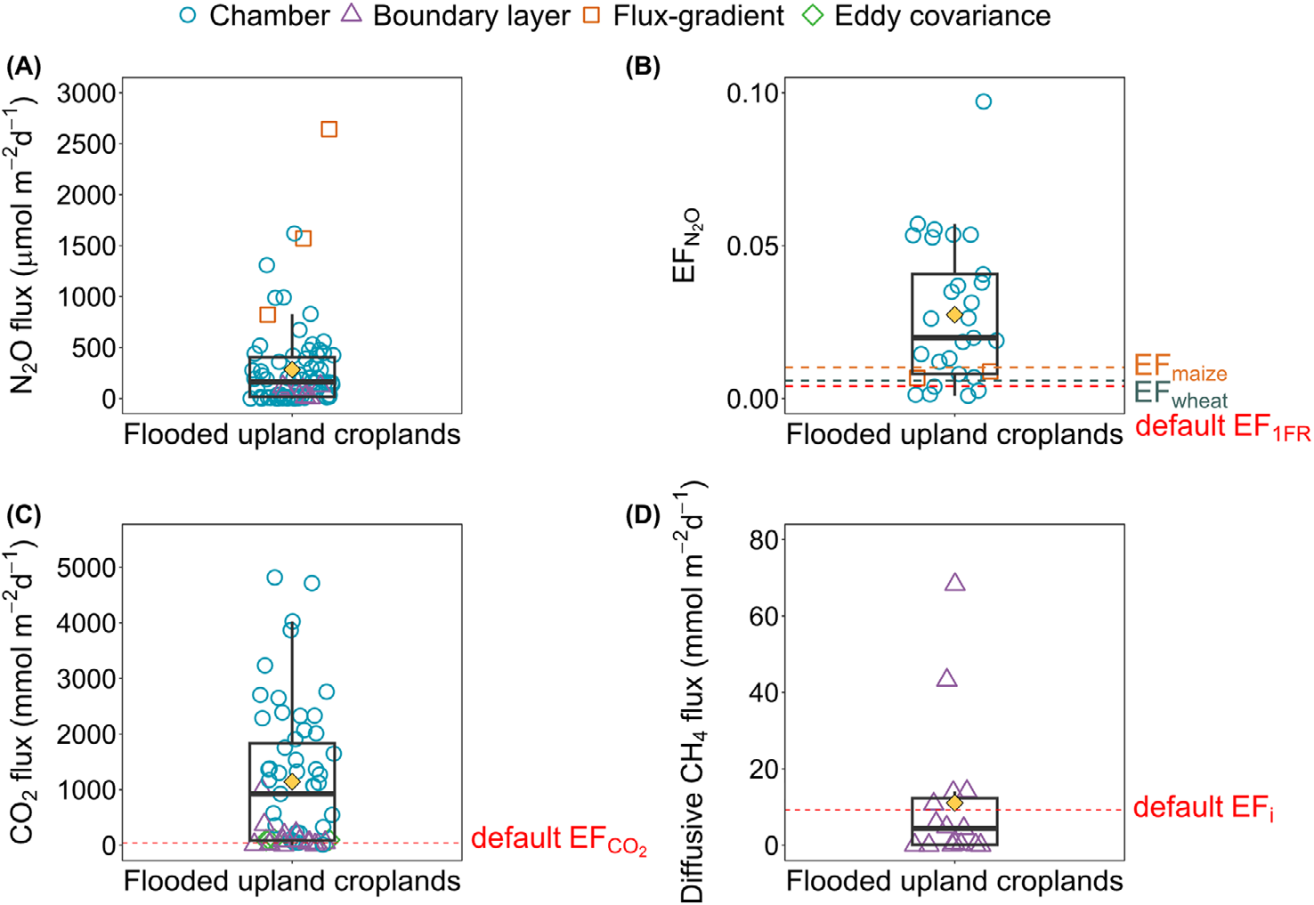
Greenhouse gas fluxes and N_2_O emission factors in flooded upland croplands. (A) N_2_O flux. (B) N_2_O emission factor (EF_N2O_). (C) CO_2_ flux. (D) Diffusive CH_4_ flux. N_2_O, CO_2_, and CH_4_ fluxes are reported on a full‐molecule basis, whereas the N_2_O emission factor is expressed on an N_2_O‐N basis. Blue circles indicate chamber measurements, orange squares indicate flux‐gradient methods, purple triangles indicate boundary layer model estimates, and green diamonds indicate eddy covariance measurements. Each point represents an individual observation, and equal weight was given to all observations when calculating the distributions and arithmetic means. The N_2_O flux panel includes 79 values: 65 chamber measurements, 11 boundary layer model estimates, and 3 flux‐gradient estimates. The N_2_O emission factor panel includes 29 values: 27 chamber measurements and 2 flux‐gradient estimates. The CO_2_ flux panel includes 59 values: 40 chamber measurements, 15 boundary layer model estimates, and 4 eddy covariance measurements. The CH_4_ flux panel includes 15 values, all of which are boundary layer model estimates. Boxes show the interquartile range, center lines show medians, yellow diamonds show means and whiskers extend to 1.5 times the interquartile range. Red dashed lines indicate Intergovernmental Panel on Climate Change (IPCC) default values for N_2_O from flooded paddies (0.004), CO_2_ from flooded lands (41.2 mmol CO_2_ m^−2^ d^−1^) and CH_4_ from flooded paddies (9.2 mmol CH_4_ m^−2^ d^−1^). Orange and green dashed lines in (B) indicate global N_2_O emission factors for maize (0.0102) and wheat (0.0058), respectively, during 1980–2018 [24].

Temporary flooding was associated with high emissions of all three gases rather than affecting a single pathway. The observed N_2_O emission factor was nearly 7 times the IPCC default for flooded rice paddies [20], whereas mean CO_2_ fluxes were approximately 28 times the default for newly flooded lands [19]. Together, these results indicate that temporarily flooded upland croplands can act as an overlooked agricultural source of GHG emissions.

### 2.2 Environmental Drivers Reveal Different Controls

The three GHGs showed distinct relationships with environmental variables, indicating that flooding affected multiple biogeochemical processes rather than a single emission pathway (Figure 3). N_2_O fluxes were negatively correlated with soil dissolved organic carbon (DOC) concentration (Figure 3A) and the DOC:NO_3_^−^ ratio (Figure S2A), but positively correlated with soil nitrate availability (Figure S2B). By contrast, CO_2_ fluxes decreased significantly with increasing water pH (Figure 3B). Diffusive CH_4_ fluxes showed no significant relationship with dissolved oxygen (DO) (Figure 3C). These results show that flooding imposes distinct environmental controls on different GHGs rather than producing a uniform response across gases.

**FIGURE 3:**
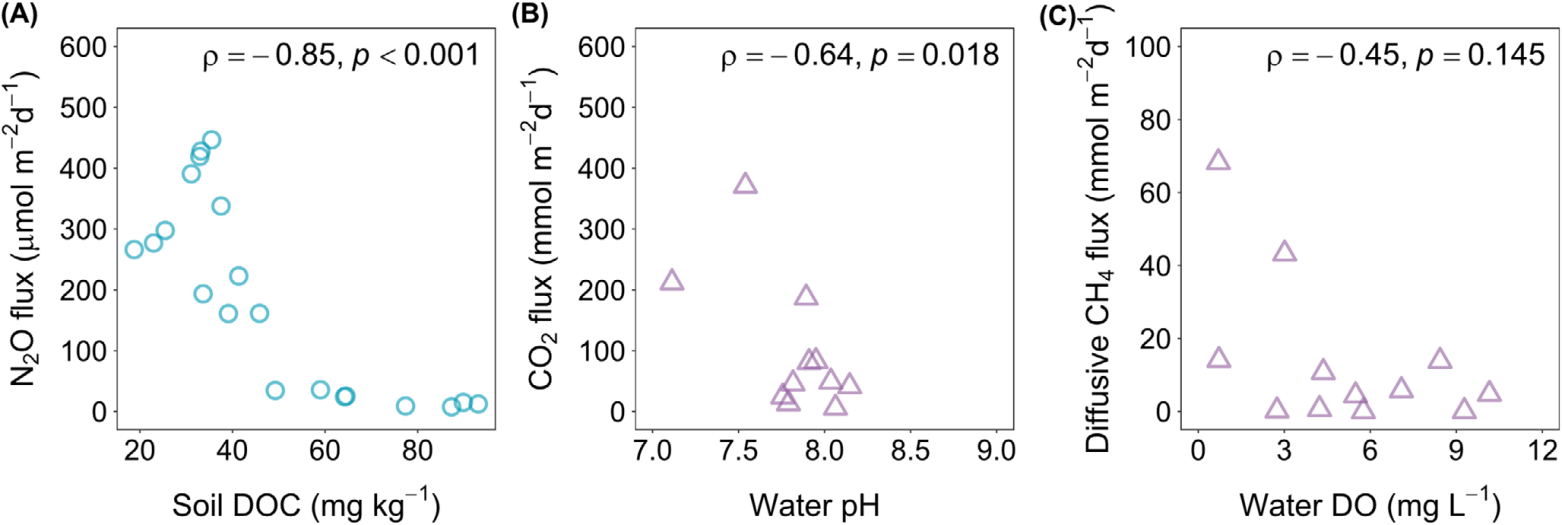
Relationships between greenhouse gas fluxes and environmental drivers during flooding. (A) N_2_O flux versus soil dissolved organic carbon (DOC). (B) CO_2_ flux versus water pH. (C) Diffusive CH_4_ flux versus dissolved oxygen (DO). Blue circles indicate chamber measurements and purple triangles indicate boundary‐layer model estimates. Spearman's rank correlation coefficients (ρ) and two‐sided *p*‐values are shown in each panel.

### 2.3 Emissions Evolve Across Flooding Phases

Flood‐associated GHG emissions extended beyond visible inundation and varied across the pre‐flooding, flooding, and drainage phases (Figure 4). Before complete flooding, N_2_O and CO_2_ fluxes increased as soil water content rose (Figure 4A,B). During flooding, these fluxes remained elevated, and after drainage they declined but frequently remained above pre‐flooding levels (Figure 4A,B). Diffusive CH_4_ fluxes showed a tendency to increase with flooding duration during the flooding phase (Figure 4C). However, CH_4_ observations were sparse during the pre‐flooding and drainage phases, which constrained our ability to characterize CH_4_ dynamics during these transitional periods.

**FIGURE 4:**
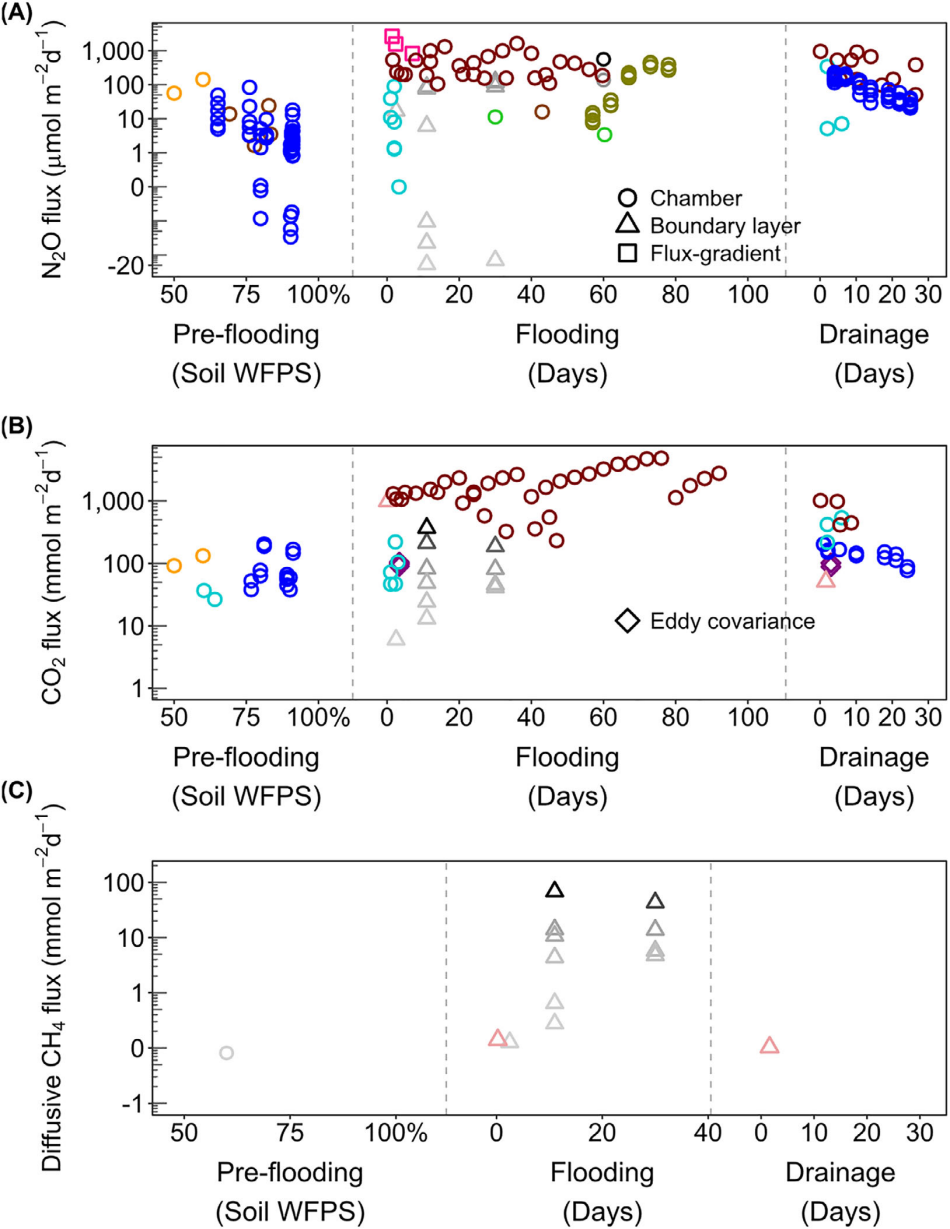
Temporal dynamics of greenhouse gas emissions during flooding in upland croplands. (A) N_2_O flux. (B) CO_2_ flux. (C) Diffusive CH_4_ flux. The *x*‐axis represents the pre‐flooding, flooding, and drainage phases, described by soil water‐filled pore space (WFPS), flooding duration, and drainage duration, respectively. The numbers of observations during the pre‐flooding, flooding, and drainage phases were 54, 73, and 56 for N_2_O; 18, 55, and 24 for CO_2_; and 1, 12, and 1 for CH_4_, respectively. Circles indicate chamber measurements, triangles indicate boundary‐layer model estimates, squares indicate flux‐gradient methods and diamonds indicate eddy covariance measurements. Colors indicate individual study sites. All data shown in this figure were obtained from the global literature synthesis. The *y*‐axis uses a symmetric pseudo‐logarithmic scale that preserves the sign of each value. It is continuous and approximately linear near zero but increasingly compresses larger absolute values. Zero values remain at zero, positive fluxes representing net emissions are plotted above zero, and negative fluxes representing net uptake are retained below zero.

These observations show that flood‐associated GHG emissions span the full flooding cycle rather than being confined to standing‐water conditions. Emissions observed before flooding and after drainage (Tables S2 and S3) indicate that estimates based only on visible inundation are likely to underestimate total flood‐associated GHG emissions. However, only one CH_4_ observation was available in each of the pre‐flooding and drainage phases, preventing a robust assessment of their contributions to the global CH_4_ budget.

### 2.4 Flooded Croplands are a Substantial Global Source

Upscaling site‐level diffusive fluxes with observed flooding duration and satellite‐derived flood‐exposed cropland area yielded an estimated 63.3 Tg CO_2_‐eq emissions per year from temporarily flooded upland croplands during the standing‐water periods (Table 1). This total comprised 7.2 Tg from N_2_O, 34.6 Tg from CO_2_ and 21.5 Tg from CH_4_. The calculation used a mean flooding duration of 23 days and a mean annual flood‐exposed upland cropland area of 15.9 Mha, indicating that episodic flooding represents a substantial GHG source from global upland agriculture.

**TABLE 1:** Estimated global diffusive greenhouse gas emissions from temporarily flooded upland croplands during flooding periods.

GHG	Diffusive flux (mmol m^−2^ d^−1^)	Flooding duration (days)	Flooding area (Mha)	Diffusive emission (Tg CO_2_‐eq yr^−1^)
N_2_O	0.2 (0.05–0.4)	23 (14–31)	15.9 (12.1–19.9)	7.2 (1.7–17.2)
CO_2_	217.1 (69.3–470.7)	23 (14–31)	15.9 (12.1–19.9)	34.6 (9.0–83.7)
CH_4_	13.8 (4.2–26.4)	23 (14–31)	15.9 (12.1–19.9)	21.5 (6.2–45.6)

*Note*. Values are means with 95% uncertainty intervals in parentheses. For the flux values used in global upscaling, repeated observations were first averaged within each site, after which equal weight was given to each site. Global emissions were estimated by combining site‐level mean fluxes, flooding duration and satellite‐derived flooding cropland area. Carbon dioxide‐equivalent emissions (CO_2_‐eq) were calculated using 100‐year global warming potentials. GHG, greenhouse gas; Mha, million hectares.

Uncertainty analysis showed substantial variation in the estimated emissions (Table 1), reflecting uncertainties in site‐level fluxes, flooding duration, and flood‐exposed cropland area. Nevertheless, emissions remained substantial across the 95% uncertainty intervals for all three gases, indicating that this source persists across the uncertainty range.

## 3 Discussion

Temporarily flooded upland croplands have received limited attention in global GHG research because they are generally treated as aerobic agricultural systems rather than flood‐prone ecosystems. Our results qualify this assumption by showing that episodic flooding can rapidly transform upland croplands into transient sources of N_2_O, CO_2_ and CH_4_. The magnitude of these emissions indicates that this process warrants consideration in agricultural GHG inventories. Rather than representing isolated responses to extreme rainfall, temporary flooding can constitute an overlooked biogeochemical process with implications for regional and global GHG budgets.

The emissions observed during flooding are consistent with a rapid reorganization of carbon and nitrogen cycling following oxygen depletion. Before inundation, upland croplands commonly accumulate nitrate through fertilization and aerobic nitrification [26]. Once soils become saturated, denitrification can replace nitrification as the dominant nitrogen transformation pathway [27], generating N_2_O while carbon availability regulates the extent to which N_2_O is further reduced to N_2_ [8, 28]. This mechanism is consistent with the observed relationships between N_2_O emissions, dissolved organic carbon (Figure 3A) and nitrate availability (Figure S2B), indicating that substrate stoichiometry influences denitrification efficiency (Figure S2A) [29].

In parallel, flooding can enhance microbial decomposition of flood‐mobilized organic matter, crop residues and roots [30], while carbonate dissolution under acidified conditions may provide an additional source of CO_2_ [31, 32]. Persistent anaerobic microsites can also promote methanogenesis [33], whereas limited aerenchyma development in upland crops probably restricts oxygen transport to the rhizosphere and suppresses methane oxidation relative to flooded rice paddies [34]. These responses indicate that flooding activates multiple interacting carbon and nitrogen pathways, explaining why different GHGs exhibit distinct environmental controls rather than a common response [35, 36].

Flood‐associated emissions cannot be represented adequately by measurements collected only during visible inundation. Elevated N_2_O and CO_2_ emissions before complete flooding indicate that biogeochemical activity begins during the transition from aerobic to anaerobic conditions [37], whereas sustained emissions after drainage suggest that recovery toward aerobic conditions is also an important phase of the emission cycle [12, 13]. Although observations of CH_4_ before flooding and after drainage remain limited, available evidence indicates that methane emissions may persist beyond periods of standing water [38]. These findings suggest that visible flooding duration alone is unlikely to capture the full magnitude of flood‐associated GHG emissions and highlight the importance of monitoring complete flooding sequences, from rainfall onset to post‐flood drainage.

The global implications of these findings extend beyond the magnitude of the estimated emissions. The 63.3 Tg CO_2_‐eq yr^−1^ estimated for standing‐water flooding periods is equivalent to approximately 0.8% of global farm‐gate crop and livestock emissions in 2023 (8.1 Gt CO_2_‐eq) [39] and 2.5% of estimated global cropland emissions circa 2020 (2.5 Gt CO_2_‐eq yr^−1^) [4]. These comparisons indicate the potential magnitude of this source, although the estimates differ in reference year, source coverage, and treatment of land CO_2_ fluxes. Current IPCC methodologies explicitly consider flooded rice paddies and newly flooded lands but do not include temporarily flooded upland croplands [19, 20, 21]. This omission has probably received limited attention because flooding has often been viewed as an exceptional disturbance rather than a recurring component of upland agricultural systems. However, climate change is increasing extreme rainfall and flood occurrence across many agricultural regions [1, 2, 3]. The IPCC assesses that heavy precipitation in East Asia will become more frequent and intense [40]. An analysis based on Phase 6 of the Coupled Model Intercomparison Project a 127%–131% increase in annual rainfall accumulated on extremely wet days in North China by 2081–2100 under the Shared Socioeconomic Pathway (SSP) 5‐8.5 scenario, relative to 1995–2014 [41]. Another analysis estimates annual probabilities of 3.9% under SSP2‐4.5 and 5.1% under SSP5‐8.5 for a regional maximum one‐day rainfall event comparable to that of 2023 during 2041–2060 [42]. Under these conditions, temporary flooding may become an increasingly important regulator of agricultural GHG emissions. Our observation‐based estimate therefore suggests not only that an additional emission source exists, but also that current agricultural GHG inventories may underestimate emissions associated with climate extremes.

Our global estimate should be regarded as conservative. The present analysis excludes emissions before flooding and after drainage, does not account for methane ebullition, and may underestimate flood extent because shallow or short‐lived inundation is not always detected by satellite products [23, 43, 44]. Available observations also remain geographically uneven, particularly in tropical regions and for CH_4_ measurements. Our field survey in the North China Plain provides regional evidence, while the literature synthesis broadens the coverage across crops and regions. However, the estimate of 63.3 Tg CO_2_‐eq yr^−1^ was derived by extrapolating these observations using the flooded cropland area and mean flooding duration of the standing‐water flooding periods. It therefore does not represent a directly measured global flux.

A temporal sensitivity analysis based on studies with repeated measurements showed that alternative flooding durations yielded estimates ranging from 39.2 to 86.7 Tg CO_2_‐eq yr^−1^, compared with 63.3 Tg CO_2_‐eq yr^−1^ under the mean‐duration assumption (Table S5). Sparse observations precluded robust global estimates for individual crops or regions. As a complementary crop‐specific sensitivity test, restricting the analysis to maize observations increased the Monte Carlo estimate from 63.3 to 74.7 Tg CO_2_‐eq yr^−1^, an increase of approximately 18% (Table S6). These limitations affect the magnitude of the global estimate and highlight the need for broader observations across regions and gases. Future studies combining continuous flux measurements with improved flood mapping across diverse climatic regions should reduce these uncertainties and provide a stronger basis for incorporating flood‐driven emissions into agricultural GHG inventories and Earth system models.

## 4 Conclusion

Collectively, our findings show that temporary flooding alters the biogeochemical functioning of upland croplands and can create a previously overlooked pathway of agricultural GHG emissions. The estimate of 63.3 Tg CO_2_‐eq yr^−1^ applies only to the standing‐water flooding period and excludes emissions pre‐flooding and during drainage. The global magnitude of this pathway remains also uncertain because the estimate assumes that the measured fluxes represent average conditions throughout the entire flooding period. In addition, both the field survey and literature synthesis were based on flood events that had already occurred; neither dataset provides an estimate of flooding probability at the sampled fields. As extreme rainfall events become more frequent under future climate change, representing flood‐driven emissions in agricultural inventories is important for improving global GHG accounting and constraining climate feedbacks. The magnitude of these emissions further underscores the significance of temporary flooding as an agricultural GHG source that cannot be ignored and may inform future IPCC assessments.

## 5 Methods

### 5.1 Field Observations

Field observations were conducted after natural flooding events at 11 maize fields across the North China Plain, one of the major agricultural regions in China, which is characterized by a temperate monsoon climate and intensive cereal production [45]. Floodwater samples were collected from ten maize‐field sites following a regional flood in August 2023 and from one additional site following a separate, smaller flood in August 2025. The 2023 rainfall event was exceptional [46], an independent analysis estimated a return period of approximately 74 years for both the regional one‐day and five‐day rainfall maxima [42]. Rainfall characteristics, flooding depth and flooded area for each event are summarized in Table S1. The North China Plain was selected because it is one of the world's largest intensive cereal‐producing regions and experiences frequent pluvial flooding, making it a relevant case study for flood‐prone upland agricultural systems.

At each site, floodwater overlying the soil surface was sampled for GHG and water chemistry analyses. Dissolved N_2_O, CO_2_, and CH_4_ concentrations were determined using headspace equilibration followed by gas chromatography. Dissolved organic carbon (DOC), ammonium (NH_4_^+^–N), nitrate (NO_3_^−^–N), dissolved oxygen (DO), and pH were measured. Meteorological data were obtained from the China Meteorological Administration. Detailed procedures for headspace equilibration, water sampling, sample preservation, and the analyses of dissolved organic carbon, ammonium, nitrate, dissolved oxygen, and pH are provided in Methods S1.

Diffusive GHG fluxes were calculated from air–water concentration gradients using gas transfer velocity (k) parameterizations. Three commonly used models of gas transfer velocity normalized to a Schmidt number of 600 (k_*600*_) Raymond and Cole (2001; RC01) [47], Cole and Caraco (1998; CC98) [48], and Crusius and Wanninkhof (2003; CW03) [49]—were evaluated against independent floating chamber measurements collected from flooded maize fields on the North China Plain. The CC98 parameterization showed the closest agreement with chamber‐derived fluxes across N_2_O, CO_2_, and CH_4_ and was therefore adopted for all subsequent flux calculations. Diffusive flux calculations and Schmidt‐number normalization are described in Methods S2 and Equations (S1) and (S2). The comparison of the RC01, CC98, and CW03 parameterizations and the rationale for selecting CC98 are provided in Methods S3 and Figure S1.

Dissolved gas concentrations were calculated from headspace concentrations using Henry's law and published gas‐solubility functions [50, 51]. Diffusive fluxes were then estimated from air–water concentration gradients following the boundary‐layer framework [52], with gas‐specific transfer velocities normalized to a Schmidt number of 600 according to Wanninkhof [53].

### 5.2 Global Literature Synthesis

A global database of GHG emissions from temporarily flooded upland croplands was compiled through a systematic literature survey of the Web of Science and China National Knowledge Infrastructure. Studies published before 31 May 2025 were screened for field measurements of GHG emissions following natural flooding events in upland cropping systems. Studies were included only if they (i) reported in situ measurements during naturally occurring flooding events, (ii) focused on upland crops rather than permanently flooded agricultural systems and (iii) presented directly measured GHG fluxes. Mesocosm experiments, laboratory incubations and model‐only studies were excluded. Following screening, the literature database comprised 23 study sites from 16 independent studies distributed across Asia, Europe, North America and Australia (Figure 1). The literature synthesis was treated as a separate observational component from the North China Plain field survey. Its purpose was to broaden the evidence base across crops, geographic regions, flooding conditions, and measurement methods, although the resulting coverage remained uneven among gases, crops, and regions. Combined with the field observations, the dataset contained 79, 59 and 15 flux measurements for N_2_O, CO_2_ and diffusive CH_4_, respectively, together with 86 flooding‐duration observations.

For each study, GHG fluxes (N_2_O, CO_2_ and CH_4_), geographic location, climatic conditions, soil and water physicochemical properties, and flooding characteristics were extracted where available. Specifically, the compiled database included latitude, longitude, mean annual temperature, mean annual precipitation, elevation, soil or water temperature, water‐filled pore space (WFPS), dissolved oxygen (DO), pH, dissolved organic carbon (DOC), dissolved inorganic carbon, ammonium (NH_4_^+^–N), nitrate (NO_3_^−^–N), flooding depth. The reported durations of the pre‐flooding, flooding, and drainage phases were summarized for each site (Table S4). These phase‐specific durations were used to position the measurements within the flood cycle when exact timing relative to rainfall was unavailable [54]. When numerical values were not reported in the text or tables, data were digitized from published figures using WebPlotDigitizer. GHG fluxes were measured using static chambers, dynamic chambers, flux‐gradient methods or eddy covariance techniques.

### 5.3 Estimation of Global Greenhouse Gas Emissions

Flood‐associated GHG emission factors were estimated by integrating daily fluxes over the observed flooding duration, as detailed in Methods S4. The N_2_O emission factor was calculated according to Equation (S3), following the IPCC Tier 1 framework as the ratio of cumulative N_2_O‐N emissions to the nitrogen application rate [20]. Estimated N_2_O emission factors were compared with the corresponding IPCC default value for flooded rice paddies and previously reported global emission factors for maize and wheat [24]. Mean CO_2_ and CH_4_ fluxes were compared with the corresponding IPCC default values for newly flooded lands and flooded rice paddies, respectively [19, 21].

Global diffusive GHG emissions from temporarily flooded upland croplands were estimated by combining site‐level mean GHG fluxes, flooding duration and flood‐exposed cropland area. To avoid disproportionate weighting of intensively sampled locations, repeated observations within each site were averaged before global upscaling, yielding 19, 16 and 12 site‐level mean flux values for N_2_O, CO_2_ and diffusive CH_4_, respectively. Each site therefore received equal weight, irrespective of the number of repeated observations. These values differ from the means from all individual observations because repeated measurements did not increase the weight of a site in the global estimate. Flood‐exposed cropland area was estimated by intersecting annual flood maps from the Global Flood Database (2000–2018) [23] with rainfed harvested‐area maps for wheat, maize, barley, soybean and sugarcane from SPAM 2020 v2.0 [55]. Pixels were classified as target upland croplands when the summed harvested area of these five crops was greater than zero.

Global extrapolation was conducted only after the field and literature records had been harmonized and repeated measurements had been averaged within each site. To assess the influence of temporal upscaling, we repeated the calculations using alternative flooding durations derived from studies with repeated measurements. The resulting estimate therefore represents emissions during the flooding duration under the assumptions used for temporal and spatial upscaling.

### 5.4 Uncertainty Analysis

Uncertainty in global GHG emission estimates was quantified using non‐parametric bootstrap resampling and Monte Carlo propagation. To account for unequal sampling intensity among study sites, repeated observations within individual sites were first averaged to generate site‐level mean values. Separate bootstrap distributions were then generated for site‐level mean GHG fluxes, site‐level mean flooding duration, and annual flood‐exposed cropland area using 10 000 resampling iterations [56].

Overall uncertainty was propagated using Monte Carlo simulation [57]. During each iteration, one value was randomly sampled from the bootstrap distributions of site‐level mean GHG fluxes, flooding duration, and annual flood‐exposed cropland area. These input variables were sampled independently because information on their covariance was not available, and total GHG emissions were recalculated for each iteration. The Monte Carlo mean therefore represents an observation‐based estimate from limited and heterogeneous data, not a directly observed global flux. The Monte Carlo simulation was repeated 10 000 times for each gas to generate the uncertainty distribution of global GHG emissions.

Final global GHG emissions are reported as the Monte Carlo mean with corresponding 95% confidence intervals. Carbon dioxide‐equivalent emissions (CO_2_‐eq) were calculated using 100‐year global warming potentials of 273 for N_2_O and 27 for CH_4_, following the IPCC Sixth Assessment Report.

### 5.5 Statistical Analysis

Statistical analyses were performed in R (version 4.3.1). GHG fluxes and N_2_O emission factors are presented as mean ± standard deviation unless otherwise stated. Summary statistics were calculated from individual observations, with each observation receiving equal weight. For global upscaling, repeated observations were first averaged within each site, after which each site received equal weight. Relationships between GHG fluxes and environmental variables were evaluated using Spearman's rank correlation, and corresponding correlation coefficients (ρ) and two‐sided *p*‐values are reported. Missing values were not imputed. No observations were excluded as outliers. Bootstrap resampling was used to estimate confidence intervals for global emission estimates, whereas Monte Carlo simulation was used to propagate uncertainty during global upscaling. Statistical significance was defined as *p* < 0.05. Data visualization was performed using ggplot2 in R.

## Author Contributions

S.B. developed the methodology, performed formal analyses, and wrote the original draft. Z.Y. conceptualized and supervised the study and revised the manuscript. Z.G. contributed to investigation. S.‐L.L., G.Y., C.‐Q.L., H.T. and P.S. contributed to the manuscript revision. All authors discussed the results and approved the final manuscript.

## Funding

This work was funded by the Fundamental and Interdisciplinary Disciplines Breakthrough Plan of the Ministry of Education of China (JYB2025XDXM907) and the National Natural Science Foundation of China (42293262).

## Conflicts of Interest

The authors declare no conflicts of interest.

## Supporting information




**Supporting File**: advs78387‐sup‐0001‐SuppMat.docx.

## Data Availability

The data and code supporting the findings of this study are openly available in *Zenodo* at https://doi.org/10.5281/zenodo.23343729. Publicly available source datasets used in this study are available from their original repositories, as cited in the article.
