# Supplementary material for: Flooding‐Driven Greenhouse Gas Emissions From Global Upland Croplands
Source: Adv Sci (Weinh). 2026 Oct 26:e78387. Online ahead of print. doi: 10.1002/advs.78387 (PMC13710713; doi:10.1002/advs.78387)
Supplement: Supplementary file 1 [file ADVS-9999-e78387-s001.docx]

**Supporting Information**

**Flooding-Driven Greenhouse Gas Emissions from Global Upland Croplands**

*Shuilian Bi, Zhifeng Yan*, Zhengkui Ge, Si-Liang Li, Guirui Yu, Cong-Qiang Liu, Hanqin Tian, and Pete Smith*

*Corresponding author: yanzf17@tju.edu.cn (Z.Y.)

**This Supporting Information Includes:**

• Supplementary Methods

• Figures S1 and S2

• Tables S1 to S6

• Numbered references follow the combined reference list of the main manuscript; the Supporting Information-only reference is listed at the end.

**Supplementary Methods**

**S1. Field Observations and Sample Collection**

Dissolved greenhouse gas (GHG) concentrations in the overlying water were collected by the headspace equilibrium method. Water samples were collected at 15 cm below the water surface using 75 mL serum bottles and sealed underwater with butyl rubber stoppers and aluminum caps. Ultrahigh-purity N_2_ (99.999%, 20 mL) was then injected to create a headspace, and the bottles were shaken to allow gas equilibration between the water and headspace. Subsequently, gas samples were analyzed using a gas chromatograph (Agilent 7890B GC-ECD, USA) to determine GHG concentrations.

Overlying water samples for water chemistry analysis were collected at a depth of 15 cm using 500 mL polyethylene bottles. Samples were immediately filtered through 0.70 μm Whatman GF/F filters and preserved at -20 °C prior to analysis within two weeks. Dissolved organic carbon (DOC) was analyzed with a TOC analyzer (TOC-L, Shimadzu, Japan). Ammonium (NH_4_^+^–N) and nitrate (NO_3_^-^–N) concentrations were quantified using a flow injection analyzer (SKALAR, The Netherlands). In situ measurements of dissolved oxygen (DO) and pH were made using a portable multiparameter probe (YSI EXO1, USA). Meteorological variables were retrieved from the China Meteorological Administration (http://data.cma.cn).

**S2. Diffusive Flux Calculation**

Dissolved GHG concentrations in surface water were calculated from headspace GHG concentrations based on Henry’s law and published gas-solubility functions.^[50,51]^ Diffusive GHG fluxes were calculated as Equation (S1):

$Flux=k\times\left( C_{w}-C_{eq} \right)$	(S1)

where *Flux* is the diffusive GHG flux across the air-water interface, *k* is the gas transfer velocity (cm h^-1^), *C_w_* is the measured concentration of the gas in surface water (μmol L^-1^), and *C_eq_* is the concentration of the gas in surface water in equilibrium with the atmosphere under *in-situ* conditions (μmol L^-1^).

Gas transfer velocity *k* (cm h^-1^) was calculated using Equation (S2):

$k=k_{600}\times{(S_{C}/\text{600})}^{-n}$	(S2)

where *k_600_* is the gas transfer velocity normalized to a Schmidt number of 600 (cm h^-1^), S_C_ is the Schmidt number of the gas at the measured water temperature, and *n* is an empirical exponent describing the dependence of gas transfer velocity on the Schmidt number; *n* was set to 1/2 in this study following Wanninkhof.^[53]^

**S3. Rationale for Selecting the CC98 Model**

Diffusive GHG fluxes estimated using three empirical gas-transfer models—RC01,^[47]^ CC98,^[48]^ and CW03^[49]^—were compared with fluxes measured by floating chambers. All three models showed strong correlations with chamber-based measurements (R^2^ = 0.99, *p* < 0.001). However, the RC01 model tended to overestimate fluxes, whereas the CW03 model generally produced lower estimates. The CC98 model showed the closest overall agreement with the 1:1 line across N_2_O, CO_2_, and CH_4_, indicating relatively low systematic bias. Therefore, CC98 was selected for subsequent estimation of diffusive GHG fluxes.

**S4. Calculation of Greenhouse Gas Emission Factors and Global Emissions**

Emission factors for flooded upland croplands were calculated by multiplying measured daily fluxes by flooding duration. For N_2_O, the emission factor followed the Intergovernmental Panel on Climate Change (IPCC) Tier 1 framework and was defined as the ratio of cumulative N_2_O–N emissions to nitrogen application rate:^[20]^

$\text{EF = }\frac{\text{E}}{\text{N}_{\text{input}}}$	(S3)

where *E* is the cumulative N_2_O-N emission during the flooding period and *N_input_* is the nitrogen application rate. The N_2_O emission factor was compared with the IPCC default value for flooded rice paddies and with global crop-specific values for maize and wheat.^[20,24]^

**Supplementary Figures**

**Figure S1.** Comparison between floating chamber-measured greenhouse gas fluxes and fluxes estimated using the CC98 model.^[48]^ Model-derived diffusive fluxes of N_2_O, CO_2_, and CH_4_ were compared with corresponding floating-chamber measurements on a log-log scale.

**Figure S2.** Relationships between N_2_O fluxes and environmental drivers during flooding periods. Spearman correlation analyses between (A) N_2_O flux and soil DOC:NO_3_^-^ ratio and (B) N_2_O flux and soil NO_3_^-^ concentration. Individual data points indicate observations using different measurement techniques: blue circles represent chamber methods and orange squares represent flux-gradient methods. Spearman’s rank correlation coefficients (ρ) and P values are shown in each panel.

**Supplementary Tables**

**Table S1. Hydrological characteristics of flooding events in maize fields on the North China Plain following extreme rainfall (ten sites in 2023) and a smaller rainfall event (one site in 2025).**

| **Year** | **Sampling** **date** | **Rainfall** **(mm)** | **Flooding** **depth** **(cm)** | **Flooded** **area** **(km^2^)** | **Data source** |
| --- | --- | --- | --- | --- | --- |
| 2023 | 13–28 August | 153 | <30 (n = 5); 50–100 (n = 4); 170 (n = 1) | 100 | Rainfall and flooded area from Lan and Wang ^[S1]^; flooding depth from field observations in this study |
| 2025 | 20 August | 25 | 10 | — | Rainfall from China’s National Surface Weather Station Basic Meteorological Elements Dataset (V3.0; http://data.cma.cn/); flooding depth from field observation in this study |

Note. Rainfall represents cumulative precipitation during the corresponding rainfall period. Field observations were conducted once in each year, including ten sampling sites in 2023 and one sampling site in 2025. The dash indicates that flooded maize area was not quantified.

**Table S2. Estimated diffusive greenhouse gas emissions and associated uncertainties from global upland croplands during pre-flooding periods.**

| **GHG** | **Diffusive flux** **(mmol** **m^-2^ d^-1^)** | **Pre-flooding** **duration** **(days)** | **Flooding area** **(Mha)** | **Diffusive emission** **(Tg CO_2_-eq yr^-1^)** |
| --- | --- | --- | --- | --- |
| N_2_O | 0.04 (0.01–0.1) | 14 (7–23) | 15.9 (12.1–19.9) | 1.1 (0.1–2.9) |
| CO_2_ | 78.0 (31.7–112.2) | 14 (7–23) | 15.9 (12.1–19.9) | 7.7 (2.7–14.8) |

Note. Values are means, with 95% confidence intervals shown in parentheses using non-parametric bootstrap resampling with 10,000 iterations.^[56]^ Diffusive fluxes are reported on a full-molecule basis. Diffusive emissions were estimated as the product of mean diffusive flux, pre-flooding duration, and mean annual flood-exposed area; uncertainty was propagated by Monte Carlo simulation.^[57]^ Because few studies precisely measured temporal changes in wetted soil area during pre-flooding periods, flooded area was used as an approximation of wetted flooding area. Diffusive emissions are expressed as Tg CO_2_-eq yr^-1^ based on 100-year global warming potential values of 273 for N_2_O and 27 for CH_4_.

**Table S3. Estimated diffusive greenhouse gas emissions and associated uncertainties from global upland croplands during drainage periods.**

| **GHG** | **Diffusive flux** **(mmol m^-2^ d^-1^)** | **Drainage** **duration** **(days)** | **Flooding area** **(Mha)** | **Diffusive emission** **(Tg CO_2_-eq yr^-1^)** |
| --- | --- | --- | --- | --- |
| N_2_O | 0.2 (0.1–0.4) | 7 (3–12) | 15.9 (12.1–19.9) | 2.7 (0.7–6.5) |
| CO_2_ | 274.8 (88.6–511.9) | 7 (3–12) | 15.9 (12.1–19.9) | 13.5 (3.1–31.3) |

Note. Values are means, with 95% confidence intervals shown in parentheses using non-parametric bootstrap resampling with 10,000 iterations.^[56]^ Diffusive fluxes are reported on a full-molecule basis. Diffusive emissions were estimated as the product of mean diffusive flux, drainage duration, and mean annual flood-exposed area; uncertainty was propagated by Monte Carlo simulation.^[57]^ Because few studies precisely measured temporal changes in wetted soil area during drainage periods, flooded area was used as an approximation of wetted flooding area. Diffusive emissions are expressed as Tg CO_2_-eq yr^-1^ based on 100-year global warming potential values of 273 for N_2_O and 27 for CH_4_.

**Table S4. Duration of hydrological phases at sites included in the flooding cycle analysis.**

| **Site ID** | **Latitude**  **(°)** | **Longitude**  **(°)** | **Pre-flooding**  **duration**  **(days)** | **Flooding**  **duration**  **(days)** | **Drainage**  **duration**  **(days)** |
| --- | --- | --- | --- | --- | --- |
| 1 | 36.95 | 116.63 | 30 | 3 | 3 |
| 2 | 39.01 | -92.75 | 3 | — | — |
| 3 | 47.43 | 126.63 | 13 | — | 14 |
| 4 | 49.64 | -97.16 | — | 3 | — |
| 5 | 40.48 | 116.83 | — | 45 | — |
| 6 | 40.07 | -88.24 | 12 | 2 | 3 |
| 7 | 55.47 | 11.72 | — | 60 | — |
| 8 | 46.68 | 132.02 | — | 67 | — |
| 9 | 55.68 | 12.57 | — | 43 | — |
| 10 | -28.28 | 153.5 | 14 | 0.25 | 2 |
| 11 | 41.98 | -93.68 | — | 39 | 13 |
| 12 | 39.40 | 116.92 | — | 2.5 | — |
| 13 | 37.80 | 115.14 | — | 11 | — |
| 14 | 37.23 | 115.74 | — | 11 | — |
| 15 | 38.66 | 116.30 | — | 11 | — |
| 16 | 39.03 | 115.71 | — | 11 | — |
| 17 | 35.89 | 115.04 | — | 11 | — |
| 18 | 39.51 | 116.93 | — | 11 | — |
| 19 | 37.20 | 114.93 | — | 30 | — |
| 20 | 38.61 | 117.17 | — | 30 | — |
| 21 | 38.21 | 116.37 | — | 30 | — |
| 22 | 38.14 | 116.06 | — | 30 | — |
| 23 | 35.97 | 115.15 | — | — | — |

Note. Site IDs correspond to those in the original data table. Pre-flooding, flooding, and drainage duration are reported as the mean values for each site. The hydrological phases describe the overall flooding cycle at each site and were not distinguished among N_2_O, CO_2_, and CH_4_ measurements. Latitude and longitude are given in decimal degrees. A dash indicates that the duration was not reported for that phase.

**Table S5. Sensitivity of global greenhouse gas emission estimates to flooding duration.**

| **Flooding duration**  **(days)** | **Estimated emission**  **(Tg CO_2_-eq yr^-1^)** | **Change from** **the reported duration estimate**  **(%)** |
| --- | --- | --- |
| 23 | 63.3 | the reported duration estimate |
| 14 | 39.2 | -38.1 |
| 31 | 86.7 | +37.2 |

Note. Values are the means of the Monte Carlo estimates and represent global greenhouse gas emissions during flooding. The reported duration estimate was calculated using the flooding duration distribution in the compiled dataset, with a mean of 23 days. In the other scenarios, the minimum and maximum flooding durations were set at 14 and 31 days, respectively, based on studies with repeated measurements. All other input variables were held constant. Percentage changes were calculated relative to the estimate obtained using the reported duration distribution.

**Table S6. Sensitivity of greenhouse gas emission estimates to crop representation in the observational dataset.**

| **Crop selection** | **N_2_O** | **CO_2_** | **CH_4_** | **Total** |
| --- | --- | --- | --- | --- |
| All available crops | 7.2 | 34.6 | 21.5 | 63.3 |
| Maize fields only | 10.0 | 41.2 | 23.5 | 74.7 |

Note. Values are in Tg CO_2_-eq yr^-1^ and refer only to emissions during the flooding duration. Both estimates used the same flooded cropland area, flooding duration, equal weighting of site means, and Monte Carlo procedure. Only the observations used to calculate the mean fluxes differed. Totals are sums of the displayed gas estimates.

Reference note. Numbered citations in the Supporting Information follow the combined reference list of the main manuscript. S1: L. Lan and X. Wang, “Large-scale flood mapping using Sentinel-1 and Sentinel-2 imagery: Spatio-temporal analysis of the 23·7 Haihe basin-wide extreme flood,” Journal of Hydrology 653 (2025): 132777. <https://doi.org/10.1016/j.jhydrol.2025.132777>.
